# Clinical Variability of *SYNJ1*-Associated Early-Onset Parkinsonism

**DOI:** 10.3389/fneur.2021.648457

**Published:** 2021-03-25

**Authors:** Suzanne Lesage, Graziella Mangone, Christelle Tesson, Hélène Bertrand, Mustapha Benmahdjoub, Selma Kesraoui, Mohamed Arezki, Andrew Singleton, Jean-Christophe Corvol, Alexis Brice

**Affiliations:** ^1^Sorbonne Université, Institut du Cerveau—Paris Brain Institute—ICM, INSERM, CNRS, Assistance Publique Hôpitaux de Paris, Hôpital Pitié-Salpêtrière, CIC Neurosciences, Paris, France; ^2^Frantz Fanon Hospital, CHU Blida, Blida, Algeria; ^3^Laboratory of Neurogenetics, National Institute on Aging, National Institutes of Health, Bethesda, MD, United States

**Keywords:** Parkinson's disease, SYNJ1, autosomal recessive inheritance, early-onset parkinsonism, atypical Parkinson's disease

## Abstract

Autosomal recessive early-onset parkinsonism is clinically and genetically heterogeneous. Mutations of three genes, *PRKN, PINK1*, and *DJ-1* cause pure phenotypes usually characterized by levodopa-responsive Parkinson's disease. By contrast, mutations of other genes, including *ATP13A2, PLA2G6, FBXO7, DNAJC6, SYNJ1, VPS13C*, and *PTRHD1*, cause rarer, more severe diseases with a poor response to levodopa, generally with additional atypical features. We performed data mining on a gene panel or whole-exome sequencing in 460 index cases with early-onset (≤ 40 years) Parkinson's disease, including 57 with autosomal recessive disease and 403 isolated cases. We identified two isolated cases carrying biallelic mutations of *SYNJ1* (double-heterozygous p.D791fs/p.Y232H and homozygous p. Y832C mutations) and two siblings with the recurrent homozygous p.R258Q mutation. All four variants were absent or rare in the Genome Aggregation Database, were predicted to be deleterious on *in silico* analysis and were found to be highly conserved between species. The patient with both the previously unknown p.D791fs and p.Y232H mutations presented with dystonia-parkinsonism accompanied by a frontal syndrome and oculomotor disturbances at the age of 39. In addition, two siblings from an Algerian consanguineous family carried the homozygous p.R258Q mutation and presented generalized tonic-clonic seizures during childhood, with severe intellectual disability, followed by progressive parkinsonism during their teens. By contrast, the isolated patient with the homozygous p. Y832C mutation, diagnosed at the age of 20, had typical parkinsonism, with no atypical symptoms and slow disease progression. Our findings expand the mutational spectrum and phenotypic profile of *SYNJ1*-related parkinsonism.

## Introduction

Parkinson's disease (PD), the second most frequent neurodegenerative disorder after Alzheimer's disease, affects about 2% of people over the age of 60 years. PD affects dopaminergic neurons in the substantia nigra, causing characteristic motor signs, such as bradykinesia, rigidity with resting tremor and postural instability; it also affects other brain areas, causing non-motor signs, such as olfactory dysfunction, cognitive impairment, psychiatric symptoms and autonomic dysfunction (1). PD is monogenic and caused by rare, highly penetrant mutations in 10–15% of PD patients, but most PD cases are sporadic and probably due to a combination of environmental, genetic and epigenetic factors. The last 25 years have seen great progress toward understanding the genetic basis of this disease, with the identification of disease-causing genes. At least 23 loci and 13 genes clearly linked to inherited forms of parkinsonism have been identified, including 10 causing early-onset (EO) autosomal recessive (AR) forms (*PRKN, PINK1, DJ-1, ATP13A2, PLA2G6, FBXO7, DNAJC6, SYNJ1, VPS13C*, and *PTRHD1*) [reviewed in Lunati et al. (2)]. AR EO PD is clinically and genetically heterogeneous: it is most frequently caused by *PRKN, PINK1* and *DJ-1* mutations, particularly in patients with a positive family history and/or consanguinity, with phenotypes resembling typical levodopa-responsive EO PD and slow disease progression. However, rare mutations of *ATP13A2, PLA2G6, FBXO7, DNAJC6, SYNJ1, VPS13C*, and *PTRHD1* cause more severe disease with additional neurological signs and symptoms, such as cognitive decline, dystonia, epilepsy, pyramidal features, and a less consistent response to levodopa [reviewed in Lunati et al. (2)].

Synaptojanin 1, encoded by *SYNJ1* on chromosome 21q22.11, was first identified in 1994 as a brain-specific 145 kDa protein highly conserved throughout evolution (3). It seems to be involved in synaptic vesicle endocytosis and recycling (4, 5). Biallelic mutations of *SYNJ1* are associated with two distinct phenotypes: EO PD (PARK20) and a severe neurodegenerative disorder with intractable seizures and tauopathies (6–20). Patients with *SYNJ1* mutations therefore display highly variable phenotypes.

We performed data mining on a gene panel or whole-exome sequencing in 460 index cases with EO PD. We identified biallelic *SYNJ1* variants in a consanguineous family and two isolated cases of PD.

## Materials and Methods

In total, 460 index cases with EO [≤40 years, mean age at onset (AAO): 33.1 ± 6.9 years] parkinsonism without mutations of genes known to cause PD and related disorders were analyzed for the presence of biallelic coding (small insertions/deletions, missense, and stop-gain changes) or splice-site (± 5 base pairs from the coding exons or synonymous variants predicted to create splice defects) variants of *SYNJ1*. Index cases were recruited through the French network for the study of Parkinson's disease genetics (PDG group) and diverse collaborations with Mediterranean countries. There were 296 male and 164 female patients; most were Caucasian (*n* = 397, 86.3%), and French (*n* = 314, 79%), the others were North African (*n* = 41, 8.9%), or of other origins (*n* = 22). A family history of PD, consistent with AR transmission, was reported in 12% of the index cases (*n* = 57), 403 of the index cases were isolated cases with suspected consanguinity (*n* = 16) or without consanguinity (*n* = 387). According to the clinical diagnostic criteria of the UK Parkinson Disease Society Brain Bank (21), most of the index cases (*n* = 431) had EO typical PD, the remaining 29 patients had EO parkinsonism with some atypical features (poor response to levodopa, pyramidal signs, oculomotor disturbances, or dementia). This study was approved by the appropriate institutional review boards, and written informed consent was obtained from all participants.

We investigated *SYNJ1* mutations, by performing data mining on a customized next-generation sequencing (NGS) targeted gene panel containing 9–70 PD-associated genes, depending on the incremental version used (Supplementary Table 1), or whole-exome sequencing data obtained as previously described (22, 23) from a large cohort of patients with EO PD.

Sanger sequencing was used to confirm variants and co-segregation analyses, where possible.

## Results

### Genetic Findings

**Figure 1:**
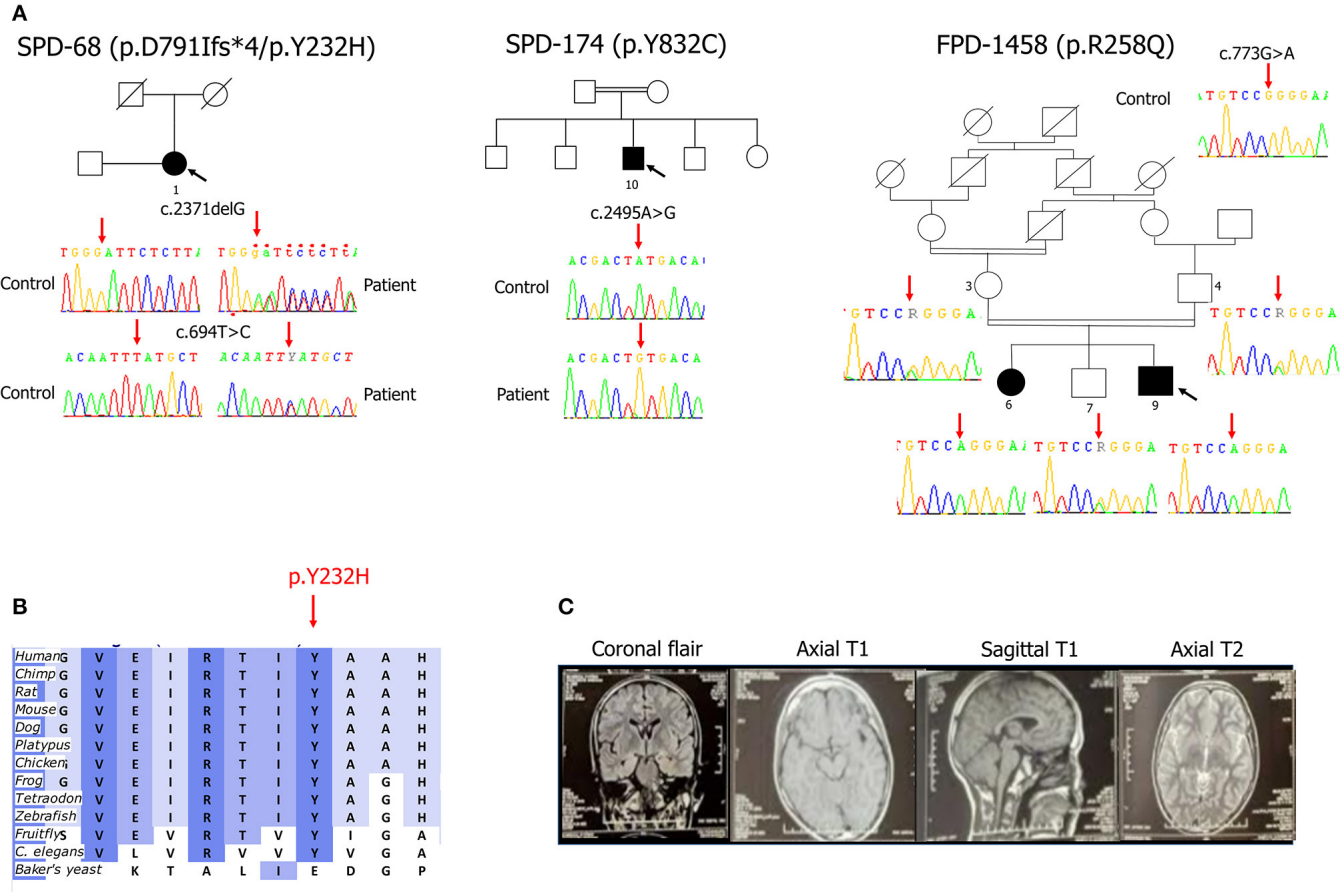
**(A)** Pedigrees of the family and the two isolated cases with early-onset Parkinson's disease carrying *SYNJ1* mutations. Affected family members are represented by black circles (female) or squares (male). The arrow indicates the index cases. Double lines indicate consanguineous parents. The corresponding Sanger sequence electrophoregrams are shown. The p.R258Q mutation segregated with the phenotype in the FPD-1458 family: the p.R258Q genotypes are highlighted by a red arrow (heterozygous state for individuals 3, 4, and 7 and homozygous for the two affected siblings, 6 and 9). **(B)** Evolutionary conservation of the regions of the p.Y232 amino-acid sequences (download from Alamut® Visual software, https://www.interactive-biosoftware.com/alamut-visual/). **(C)** Brain magnetic resonance imaging (MRI) for patient FPD-1458-9 showing the normal appearance of the various slices.

We identified a familial case (FPD-1458-9) with the recurrent homozygous missense mutation, p.R258Q (c.773G>A in exon 5) in *SYNJ1* (GenBank accession number for the longer 1,612 amino acid isoform: NM_003895.3) and two isolated cases—one consanguineous patient (SPD-174-10) carrying a homozygous missense mutation (c.2495A>G in exon 19, p.Y832C) and another patient (SPD-68-1) carrying two heterozygous mutations (a missense variant, c.694T>C in exon 5, p.Y232H and a truncating deletion, c.2371delG in exon 18, p.D791Ifs^*^4) (Figure 1A). No additional family members were available for determining parental phase for these last two mutations. The index case, FPD-1458-9, came from a consanguineous family consisting of two healthy parents, two affected and five unaffected siblings.

All four mutations were verified by Sanger sequencing. Co-segregation analyses performed in the FPD-1458 family revealed that the proband's affected sister harbored the same homozygous p.R258Q mutation, whereas both the unaffected parents and one of the five unaffected siblings for whom DNA was available were heterozygous for this mutation (Figure 1A).

No other rare homozygous or biallelic deleterious variants of PD-associated genes were identified on gene panel or whole exome analyses, for any of the three index cases.

**Figure 2:**
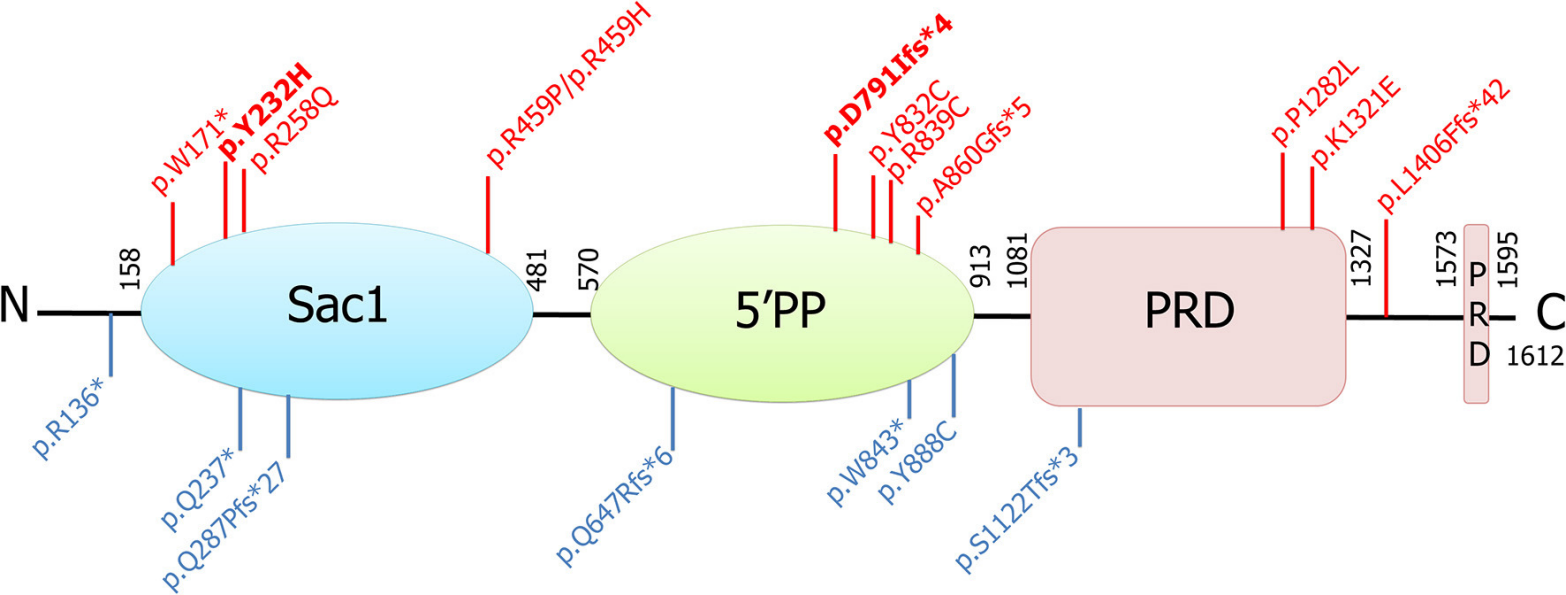
Schematic representation of the longer isoform of the synaptojanin 1 protein, its functional domains, and all associated mutations identified to date. Those found in patients with parkinsonism are shown in red (in bold, those identified in this study), and those found in patients with intractable seizures and severe neurodegeneration are shown in blue. Sac1, suppressor of actin (Sac1-like inositol domain); 5′PP, inositol-5′-phosphatase domain; PRD, prolin-rich domain.

Both the *SYNJ1* p.R258Q, and p.Y832C mutations were rare or absent from public databases and highly conserved between species (6, 7, 10). By contrast, neither of the new *SYNJ1* mutations, p.Y232H or p.D791Ifs^*^4, is present in any public database, including the Genome Aggregation Database (GnomAD). The missense variant p.Y232H is predicted to be pathogenic [SIFT: deleterious; Polyphen-2; probably damaging; MutationTaster: disease-causing; Align GVGD: Class C0 (GV: 122.78–GD: 0.00); combined annotation-dependent (CADD)_phred: 28] and has been shown to be conserved in orthologous sequences from *C. elegans* onwards (Alamut® Visual v.2.11 software, Interactive Biosoftware, Rouen, France) (Figure 1B). Both these mutations are located in functional domains: p.Y232H in the Sac1 domain and p.D791Ifs^*^4, in the 5′phosphatase domain (Figure 2).

### Clinical Outcome

#### Case Report Family FPD-1458

The two affected siblings were born to healthy first-cousin parents originating from Algeria.

The proband, FPD-1458-9, was a 25-year-old man who had suffered episodes of generalized tonic-clonic seizures at the age of 2 years after a bout of fever. These episodes were treated with sodium valproate and carbamazepine. The patient's psychomotor development was normal and he started school at the age of 6 years. Three years later, he presented a cognitive decline, leading to an interruption of his schooling. At the age of 20 years, the patient presented dysarthria, which was followed, 1 year later, by weakness of the muscles of the left arm and dystonic postures that were more marked distally and a progressive slowing of lower limb movements limiting the distance that the patient could walk. A few months later, a distal resting tremor appeared in the left upper limb, subsequently extending to the contralateral upper limb. Initial treatment with dopamine agonists was not tolerated. The patient was therefore given low doses of levodopa (50 mg), leading to a partial improvement of his motor symptoms, but rapidly resulting in disabling levodopa-induced dyskinesia. There was no evidence of autonomic dysfunction, other than urinary urgency. Clinical examination at the age of 25 years revealed a poorly cooperative patient with parkinsonism combining an intermittent resting and postural tremor in both upper limbs, head tremor, bilateral but asymmetric plastic hypertonia with cogwheel rigidity on both sides, a global motor slowing and slowing of the gait, with a reduction of the swing of the arms predominantly on the left side, slight forward camptocormia, an inexhaustible nasopalpebral reflex, excessive drooling, but no oculomotor abnormalities or pyramidal signs. Off medication, the patient had a Unified Parkinson's disease rating scale part III (UPDRS-III) score of 35/108, and a Hoehn and Yahr stage of 2.5/5. The patient's cognitive disability made it impossible to perform a cognitive evaluation. Brain magnetic resonance imaging (MRI) results were normal (Figure 1C). An electroencephalogram (EEG) showed slow activity at 5 Hz/s, with no epileptic anomalies.

The patient's 31-year-old sister (FPD-1458-6) had treated generalized epilepsy, from the age of 14–20 years. Her psychomotor development was normal until the age of 10 years when she presented cognitive decline and was unable to continue her schooling. At the age of 26 years, she displayed progressive parkinsonism with dystonia, resting tremor and upper limb postural tremor predominantly on the left side, and progressive bradykinesia. Levodopa treatment, initiated at the age of 27 years, led to a significant improvement in clinical signs, but the patient developed early levodopa-induced dyskinesia, motor fluctuations, and end-of-dose dystonia. No autonomic dysfunction was observed. Neurological examination at the age of 29 years, on “off” medication, revealed a cooperating patient with parkinsonism characterized by resting tremor in the two upper limbs, predominantly on the left, plastic hypertonia, a loss of arm swing, slow gait and facial amimia, but no oculomotor abnormalities or pyramidal syndrome. The patient had a UPDRS-III motor score of 26/108 and a Hoehn and Yahr stage of 2/5. It was not possible to perform a neuropsychological evaluation.

Other family members, including both unaffected parents and the five other unaffected siblings, displayed no signs or symptoms of epilepsy or parkinsonism.

#### Isolated Case SPD-174-10

Incomplete clinical information was obtained for patient SPD-174-10, who was lost to follow-up. This 35-year-old man was born to consanguineous parents in Senegal. He presented an extrapyramidal akineto-rigid syndrome, at the age of 20. On neurological examination at the age of 35 years, a bilateral resting tremor was observed, with no other pyramidal signs or symptoms, cerebellar syndrome, apraxia, dystonia, or oculomotor disturbances. During the “on” stage, this patient had a Hoehn and Yahr stage of 5/5 after 16 years of disease progression. No specific treatment or response to treatment was recorded, but the patient developed urinary incontinence and erectile dysfunction. No neuropsychological evaluation or brain MRI scan was recorded at his last examination.

#### Isolated Case SPD-68-1

Patient SPD-68-1 was a 61-year-old French woman, with no family history of PD or known parental consanguinity. She developed micrography, slow gait and a chin tremor at the age of 39, rapidly followed by facial and cervical dystonia. Parkinsonism was diagnosed at the age of 43. Akinesia and rigidity responded to levodopa (100 mg, 3 times/day), but with the immediate development of motor fluctuations and disabling dyskinesias. One year later, the patient presented a bilateral, asymmetric, levodopa-responsive (50%) extrapyramidal syndrome (left > right): akinesia and rigidity but no resting tremor, motor fluctuations with diphasic (facial and lower limb dystonia) and peak-dose choreic dyskinesia (oromandibular, right upper limb), permanent posterior cervical dystonia, vertical gaze palsy, freezing of gait when “off” levodopa, but no postural instability, moderate dysarthria and hypophonia. A frontal syndrome was detected with perseverations and an applause sign [Mini-Mental State Examination (MMSE) score = 27/30]. Deep tendon reflexes were brisk with no Babinski sign. There was no cerebellar dysfunction or dysautonomia. Brain computed tomography (CT) scan findings were normal. Gait freezing became more frequent, with the development of postural instability with falls 3 years after diagnosis, and a worsening of vertical gaze palsy and dysarthria. Levadopa sensitivity persisted (70% improvement following levodopa challenge, 9 years after diagnosis), but motor fluctuations and dyskinesias (diphasic and peak-dose) worsened. A moderate worsening of the frontal syndrome was observed, but instrumental functions were preserved [Mattis Dementia Rating Scale (MDRS) = 134/144 and MMSE score = 21/30, 9 years after diagnosis]. Eighteen years after diagnosis, this patient had severe dysarthria and mild dysphagia on medication.

## Discussion

*SYNJ1-*related diseases are heterogeneous in terms of their symptoms, ranging from EO typical PD (10) to EO complex parkinsonism (6–8, 10–16) (both designated PARK20), and severe neurodegeneration with intractable seizures (17–20) (Table 1).

**Table 1:** Clinical features of patients with biallelic mutations of the *SYNJ1* gene.

	**Patient**	***SYNJ1* mutations**	**Origin**	**Sex/AAE (y)**	**Transmission/consanguinity**	**Age at onset of motor symptoms (y)**	**Phenotype**	**Seizures (age at onset)**	**Response to levodopa**	**Imaging data**
**Early-onset typical or atypical parkinsonism**										
Our study	FPD-1458-9	p.R258Q (hom)	Algeria	M/25	AR/Yes	20	Parkinsonism, dysarthria, dystonia, drooling, postural instability, CI	Yes (2 y)	Partial with dyskinesia	Normal brain MRI
	FPD-1458-6	p.R258Q (hom)	Algeria	F/31	AR/Yes	26	Parkinsonism, dystonia, amimia, CI	Yes (14 y)	Good, with adverse effects (dyskinesia, motor fluctuations, dystonia)	NA
	SPD-174-10	p.Y832C (hom)	Senegal	M/35	Spo/Yes	20	Parkinsonism	No information	Unknown	NA
	SPD-68-1	p.Y232H/p.D791Ifs*4^#^ (double het)	France	F/61	Spo/No	39	Parkinsonism, facial and cervical dystonia, vertical gaze palsy, moderate dysarthria, hypophonia, postural instability, brisk deep tendon reflexes, MMSE 21/30	No	Good, with adverse effects (oromandibular and limb dystonias)	Normal brain CT scan
Krebs et al. (6)	Patient I	p.R258Q (hom)	Iran	M/29	AR/yes	20	Parkinsonism, eyelid apraxia and dysarthria at the age of 22 years, hypophonia, resting tremor, chin tremor, postural instability, no CI	Yes (3 y)	Not tolerated (severe dyskinesia)	Mild cortical atrophy, bilateral white matter hyperintensity
	Patient II	p.R258Q (hom)	Iran	F/39	AR/yes	Early twenties	Parkinsonism, right hand tremor, eyelid apraxia, severe jaw tremor, anarthria, requiring assistance to walk at the age of 32, bedbound at 37, no CI	Febrile convulsion in infancy	Not tolerated (severe dyskinesia)	Foramen magnum meningioma at the age of 37
Quadri et al. (7)	NAPO-16	p.R258Q (hom)	Italy (Sicily)	M/47	AR/yes	22	Parkinsonism, rest and action tremor, dystonia in both hands, postural instability, anarthria, severe dysarthria, eyelid apraxia and supranuclear vertical gaze palsy, dysphagia, CI	No	Not tolerated (oromandibular and limb dystonias, postural hypotension)	Diffuse cortex atrophy, hyperintensity of hippocampi, thinning midbrain quadrigeminal plate, nigrostriatal dopaminergic deficit, cortical hypometabolism
	NAPO-17	p.R258Q (hom)	Italy (Sicily)	F/31	AR/yes	28	Parkinsonism, rest and action tremor, dystonia in the hands and feet, dysarthria, dysphonia, mild dysphagia, postural instability, supranuclear vertical gaze palsy, brisk deep tendon reflexes, CI (MMSE 26/30)	No	Not tolerated (oromandibular and limb dystonias, postural hypotension)	Diffuse cortex atrophy, hyperintensity of hippocampi, thinning midbrain quadrigeminal plate, cortical hypometabolism
Olgiati et al. (8)	NAPO-41	p.R258Q (hom)	Italy (Naples)	M/31	AR/No	28	Parkinsonism, hypomimia, oromandibular tremor, trunk dystonia, impaired speech, postural instability, mild supranuclear vertical gaze limitation, drooling, dysphagia at the age of 31, MMSE 28/30	One episode (uncertain)	Not treated	No gross abnormalities, normal brain MRI, nigrostriatal dopaminergic deficit, mild bilateral hypometabolism
	NAPO-42	p.R258Q (hom)	Italy (Naples)	F/27	AR/No	26	Parkinsonism, hypomimia, oromandibular tremor, impaired speech, brisk deep tendon reflexes, MMSE 25/30	One episode (16 y)	Not treated	No gross abnormalities, normal brain MRI, nigrostriatal dopaminergic deficit, mild bilateral hypometabolism
Kirola et al. (12)	H1_1	p.R459P (hom)	India/M	M/32	AR/yes	12	Parkinsonism, drooling, dystonia, dysarthria, dysphagia, hypophonia, intense constipation, falling backwards, postural instability, no dementia	No information	Not tolerated (dyskinesia and dystonia)	Hyperintensity in substantia nigra
	H1_2	p.R459P (hom)	India/F	F/22	AR/yes	18	Parkinsonism, drooling, dystonia, dysathria, dysphagia, hypophonia, falling backwards, constipation, no dementia	No information	Not tolerated (dyskinesia and dystonia)	NA
Rauschendorf et al. (13)	Patient 1	p.W171*/p.R258Q (compound het)	Germany	M/21	AR/No	15	Generalized dystonia, Parkinsonism, severe action tremor of the tongue, head, and extremities, anarthria, CI	Yes (3–4 y)	Excellent (with L-dopa-induced dyskinesia)	Bilateral nigrostriatal dopaminergic deficit, bilateral caudate hypometabolism
	Patient 2	p.W171*/p.R258Q (compound het)	Germany	M/32	AR/No	13	Generalized dystonia, Parkinsonism, action tremor of the upper extremities, chin and tongue	First days of life	Good (no dyskinesia)	Bilateral nigrostriatal dopaminergic deficit, bilateral caudate hypometabolism
Taghavi et al. (15)	F22P1	p.R839C (hom)	Iran	M/30	AR/Yes	24	Parkinsonism, chin tremor, dysarthria, longitudinally fissured tongue, postural instability	Yes (24 y)	Poor	NA
	F22P2	p.R839C (hom)	Iran	F/47	AR/Yes	27	Parkinsonism, postural instability	No	Poor	NA
Ben Romdhan et al. (14)	PD1	p.L1406Ffs*42/p.K1321E (compound het)	Tunisia	M/23	AR/Yes	16	Parkinsonism, postural instability dystonia in the left arm, dysarthria, moderate CI (MMSE 20/30)	Yes (7 y)	Good (no dyskinesia)	Normal brain MRI
	PD2	p.L1406Ffs*42/p.K1321E (compound het)	Tunisia	F/24	AR/Yes	21	Parkinsonism, postural instability, supranuclear vertical gaze palsy, moderate CI (MMSE 21/30)	No	Good (no motor complications)	Normal brain MRI
Hong et al. (16)	II1	p.A860Gfs*5/p.P1282L (compound het)	China	F/35	AR/No	31	Parkinsonism, mild dysarthria, diplopia, dystonia, MMSE 28/30	No information	Poor	Mild cortical atrophy
	II3	p.A860Gfs*5/p.P1282L (compound het)	China	M/30	AR/No	28	Parkinsonism, diplopia, dystonia, MMSE 29/30	No information	Poor	Normal brain MRI
Xie et al. (10)	Patient 1	p.Y832C (hom)	China	F/52	AR/Yes	40	Parkinsonism, MMSE 30/30	No	Good	NA
	Patient 2	p.Y832C (hom)	China	M/54	AR/Yes	52	Parkinsonism	No	Not prescribed levodopa	NA
Kumar et al. (11)	114	p.R459H (hom)	India	No information	No information	34	Parkinsonism with poor information	No information	Unknown	NA
**Early-onset treatment-resistant seizures and severe neurodegenerative decline**										
Dyment et al. (17)	1	p.R136* (hom)	Pakistan	M/died at 6.5 years of age	Spo/Yes	NA	No parkinsonism, progressive neurodegenerative course, feeding intolerance at age of 1 year, and G tube dependence at the age of 2 years, hypotonia progressing to multiple contractures, no vocalization, cortical blindness	Yes (9 d)	Unknown	Brain MRI: mild cerebral atrophy at the age of 5 years
Hardies et al. (18)	Family A/1	p.Y888C (hom)	Morocco	F/7	AR/Yes	NA	No parkinsonism, progressive neurodegenerative course, feeding problems, hypotonia progressing to spastic tetraplegia, central visual impairment, severe intellectual disability	Yes (2.5 m)	Unknown	Normal brain MRI
	Family A/2	p.Y888C (hom)	Morocco	M/6	AR/Yes	NA	No parkinsonism, progressive neurodegenerative course, feeding problems, hypotonia progressing to spastic tetraplegia, central visual impairment, severe intellectual disability	Yes (6 m)	Unknown	Normal brain MRI
	Family B/1	p.W843* (hom)	Morocco	F/5	AR/Yes	NA	No parkinsonism, profound intellectual disability, progressive spastic tetraplegia, feeding problems with gastrostomy	Yes (1 d)	Unknown	Normal brain MRI
	Family B/2	p.W843* (hom)	Morocco	F/2.5	AR/Yes	NA	No parkinsonism, profound intellectual disability, progressive spastic tetraplegia, feeding problems with gastrostomy	Yes (1 d)	Unknown	Normal brain MRI
	Family C/1	Q647Rfs*6/p.S1122Tfs*3 (compound het)	Caucasian	M/died at the age of 2.5 years	AR/No	NA	No parkinsonism, profound intellectual disability, tube fed, dystonia	Yes (12 d)	Unknown	Normal brain MRI at age 6
	Family C/2	Q647Rfs*6/p.S1122Tfs*3 (compound het)	Caucasian	M/died at the age of 8 years	AR/No	NA	No parkinsonism, profound intellectual disability, progressive spastic tetraplegia, feeding problems with gastrostomy	Yes (1 d)	Unknown	Brain MRI: thin corpus callosum and limited gliosis and atrophy of the periventricular white matter
Al Zaabi et al. (19)	Case 1	p.Q237*(hom)	Oman	F/2	Spo/Yes	NA	No parkinsonism, profound intellectual disability, no feeding difficulties, scoliosis, significant truncal and peripheral hypotonia, and persistent palmer and plantar reflexes	Yes (2 d)	Unknown	Normal brain MRI
	Case 2	p.Q237*(hom)	Oman	M/2	First cousin of case 1/Yes	NA	No parkinsonism, microcephaly at the age of 2 years, profound intellectual disability, feeding problems, dysphagia and palatal insufficiency, head lag and axial hypotonia with hyperreflexia and clonus	Yes	Unknown	Brain MRI: mild dilation of the ventricles and subarachnoid spaces
Samanta et al. (20)	Patient 1	p.Q287Pfs*27 (hom)	Saudi Arabia	F/2	Spo/Yes	NA	No parkinsonism, profound intellectual disability, profound hypotonia, feeding problems, severe cortical visual impairment, hypotonia progressing to spastic tetraplegia, brisk deep tendon reflexes, dystonia of upper extremities	Yes (2 d)	Unknown	Normal brain MRI

*AAE, age at last examination; AR, autosomal recessive; CI, cognitive impairment; CT, computed tomography; d, day; F, female; het, heterozygote; hom, homozygote; M, male; MMSE, Mini-Mental State Examination; m, months; MRI, magnetic resonance imaging; NA, not available; Spo, sporadic; y, year*.

# *unknown parental phase*.

We report here the molecular and associated clinical findings for two familial cases with the recurrent homozygous p.R258Q mutation and two apparently sporadic cases, each carrying, either double-heterozygous of new p.D791fs/p.Y232H variants or the known homozygous p. Y832C mutation.

Two independent groups initially reported the presence of the same *SYNJ1* p.R258Q missense mutation in the homozygous state in two consanguineous sib-pairs of Sicilian and Iranian origin (6, 7). A third family from Naples, was subsequently found to have the same recurrent *SYNJ1* mutation in two non-consanguineous siblings (8). Previous haplotype analyses in the two Italian families did not support the hypothesis of a common founder for the p.R258Q variant (8), instead suggesting a possible mutational hotspot. An additional non-consanguineous family of German origin was found to carry this mutation in the heterozygous state, together with a non-sense mutation at a *trans* location (Table 1). We report here the identification of a fourth consanguineous family of Algerian origin with the homozygous p.R258Q mutation. These families were characterized by EO atypical parkinsonism, with an onset in the third decade of life, with rapid progression through the initial stages and a stabilization of the disease at later stages (24). The principal clinical features of parkinsonian disease in these patients were a combination of tremor, dystonia, bradykinesia, and, a poor response to levodopa treatment in all but our case. Additional atypical signs, such as seizures, cognitive impairment, developmental delay, and oculomotor disturbances, were variable. Indeed, our siblings presented generalized tonic-clonic seizures, as seen in the Iranian siblings, whereas the Neapolitan carriers suffered from episodes of clonic seizures. Unlike five of the other six p.R258Q carriers, our patients presented no oculomotor disturbances. Finally, mild or severe cognitive decline was observed in the Sicilian, Neapolitan and Algerian families, but not in the Iranian siblings. In both the German siblings harboring the p.R258Q variant in the compound heterozygous state, the principal clinical trait was early epilepsy followed by generalized dopa-responsive dystonia in infancy (13).

We also identified an isolated patient of Senegalese origin, from a consanguineous family, who harbored the same homozygous pY832C variant as recently reported in two Chinese consanguineous siblings with PD (10). Very little clinical information for this patient was collected at a single follow-up assessment, but the same typical parkinsonism was observed, with no atypical signs/symptoms.

Finally, we identified a non-consanguineous isolated case with two new heterozygous *SYNJ1* variants, p.Y232H and p.D791Ifs^*^4, that may be pathogenic, based on the rarity of these variants and *in silico* analyses. We thus report a case extending the age at onset for *SYNJ1* mutations carriers (39 years), identify, for the first time, *SYNJ1* mutations in apparently isolated cases. However, the parental phase of these two variants is unknown, but the phenotype of this patient with two *SYNJ1* mutations resembles that of the other PARK20 mutation carriers, who had atypical parkinsonism with early-onset disease (at 20–31 years), a rapid development of dyskinesias on levodopa, predominantly axial symptoms with rapidly progressing gait impairment and falls, oculomotor disturbances and orofacial dystonia at onset (Table 1). Parkinsonism in our case was levodopa-responsive (> 50% response after acute challenge and presence of motor fluctuations), as also reported in a few previous cases (6, 13, 14) (Table 1), but the response was difficult to evaluate in the other six patients, due to severe dyskinesia and other adverse effects (6, 7, 12). Unlike patients from most PARK20 families with atypical EO parkinsonism, this patient displayed no seizures. However, susceptibility to seizures varies considerably, even within families (14, 15). Seizures also occur in some patients with mutations of *DNAJC6* (PARK19), encoding auxilin, which has been implicated in the uncoating of synaptic vesicles, potentially resulting in alterations to synaptic vesicle recycling (25). Like auxilin, synaptojanin 1 is involved in the post-endocytic recycling of synaptic vesicles, providing additional support for the link between synaptic endosomal trafficking and PD. In addition, a link between seizures and the accumulation of tau protein has been suggested, based on the brain autopsy of a single patient with intractable epilepsy and a homozygous *SYNJ1* truncating mutation showing tau-immunoreactive neurofibrillary degeneration in the substantia nigra (17).

Synaptojanin 1 is encoded by two open-reading frames (ORFs) of 170 and 145 kDa, encoding two major isoforms of 1,612 and 1,350 amino acids, respectively. The 145 kDa ORF is strongly expressed in the brain, and the corresponding protein localizes to presynaptic nerve terminals (26). Both isoforms contain two consecutive phosphatase domains: an N-terminal Sac1-like inositol domain and a central 5′-phosphatase domain followed by a C-terminal proline-rich domain (PRD). The longer 170 kDa isoform contains an additional PRD. Interestingly, a single *SYNJ1* mutation reported by Ben Romdhan et al. (14) is located in the C-terminal domain of the longer isoform.

In total 33 *SYNJ1* mutation carriers originating from 19 families and isolated cases (13 with the PARK20-*SYNJ1* phenotype and six with infancy treatment-resistant seizures and severe neurodegenerative decline) were identified (Table 1). These last ten patients presented in the neonatal period with intractable seizures, hypotonia, feeding difficulties, and severe developmental delay but no parkinsonian signs/symptoms. These patients harbored six loss-of-function mutations (p.R136^*^, p.W843^*^, Q647Rfs^*^6, p.S1122Tfs^*^3, p.Q237^*^, p.Q287Pfs^*^27, Figure 2) in the homozygous or compound heterozygous state, shown in some cases to reduce the levels of the mutant transcript, whereas the homozygous missense mutant p.Y888C, affecting an amino acid located in the 5′-phosphatase domain of the protein, was reported to affect both the Sac1 and 5′-phosphatase activity of synaptojanin 1 (18). However, other homozygous missense mutations, such as p.R839C and p.Y832C, also affecting amino acids located in the 5′-phosphatase domain of the protein, result in typical PD or EO atypical parkinsonism, indicating that clinical severity does not depend exclusively on the protein domain affected by the missense mutations (10, 15, this study). Conversely, it could be speculated that mutations leading to premature truncation of the protein in the homozygous or compound heterozygous state, in addition to the p.Y888C mutation, may lead to severe progressive neurodegeneration, whereas homozygous missense variants or compound heterozygous variants with a missense and a premature stop variant in the *SYNJ1* gene lead to milder phenotypes associated with parkinsonism and a higher susceptibility to seizures.

In conclusion, this study has extended the mutational and clinical spectrum of *SYNJ1* associated with EO typical or atypical parkinsonism and suggests that the screening of this gene should be considered in isolated cases and in patients with a later AO.

## Data Availability Statement

The datasets presented in this study can be found in online repositories. The names of the repository/repositories and accession number(s) can be found at: www.ncbi.nlm.nih.gov/clinvar/ SCV001469064, SCV001469065.

## Ethics Statement

The studies involving human participants were reviewed and approved by INSERM, CCPPRB du Groupe Hospitalier Pitié-Salpêtrière, Paris, France and by the appropriate institutional review boards. The patients/participants provided their written informed consent to participate in this study.

## Author Contributions

SL conceived, designed and organized the study, wrote the first draft, reviewed, and critically revised the manuscript. J-CC and AB conceived the project, reviewed, and critically revised the manuscript. GM, CT, HB, MB, SK, MA, and AS contributed to the execution of the research project and critically revised the manuscript. All authors contributed to the article and approved the submitted version.

## Conflict of Interest

The authors declare that the research was conducted in the absence of any commercial or financial relationships that could be construed as a potential conflict of interest.

## Abbreviations

AAO: age at onset
AR: autosomal recessive
CADD: combined annotation-dependent depletion
EO: early-onset
GnomAD: Genome Aggregation Database
MMSE: Mini Mental State Examination
MRI: magnetic resonance imaging
NGS: next-generation sequencing
PD: Parkinson's disease
UPDRS-III: Unified Parkinson's Disease Rating Scale part III.
