# Supplementary material for: Clinical Variability of SYNJ1-Associated Early-Onset Parkinsonism
Source: Front Neurol. 2021 Mar 25;12:648457. doi: 10.3389/fneur.2021.648457 (PMC8027075; doi:10.3389/fneur.2021.648457)
Supplement: Supplementary file 1 [file Table_1.DOCX]

**The French clinicians’ network for Parkinson’s disease genetics (the PDG group) members:**

Yves Agid (site investigator, Department for the Central Nervous System, Paris), Mathieu Anheim (site investigator, Department of Neurology, Strasbourg), Michel Borg (site investigator, Department of Neurology, Nice), Alexis Brice (site investigator, Department of Genetics and Cytogenetics, Paris), Emmanuel Broussolle (site investigator, Pôle des Spécialités Neurologiques, Lyon), Jean-Christophe Corvol (site investigator, Center for Clinical Investigations, Paris), Philippe Damier (site investigator, Department of Neurology, Nantes), Luc Defebvre (site investigator, Service de Neurologie et Pathologie du Mouvement, Clinique Neurologique, Hôpital Roger Salengro, Lille), Alexandra Dürr (site investigator, Department of Genetics and Cytogenetics, Paris), Franck Durif (site investigator, Department of Neurology A, Clermont-Ferrand), Jean Luc Houetto (site investigator, service de neurologie, CHU de Poitiers, Poitiers), Paul Krack (site investigator, Pôle Psychiatrie et Neurologie, Grenoble), Stephan Klebe (site investigator, Centre for Clinical Investigations, Paris), Suzanne Lesage (site investigator, ICM INSERM U1127, Paris), Ebba Lohmann (site investigator, Department of Genetics and Cytogenetics, Paris), Maria Martinez (site investigator, INSERM Unit 563, Toulouse), Graziella Mangone (site investigator, Centre for Clinical Investigations, Paris), Pierre Pollak (site investigator, Pôle Psychiatrie et Neurologie, Grenoble), Olivier Rascol (site investigator, Clinical Investigation Centre, Toulouse), François Tison (site investigator, Pôle des Neurosciences, Cliniques de Neurologie, Bordeaux), Christine Tranchant (site investigator, Department of Neurology, Strasbourg), Marc Vérin (site investigator, Department of Neurology, Rennes), François Viallet (site investigator, Department of Neurology, Aix-en-Provence), and Marie Vidailhet (site investigator, Department of Neurology, Paris).

**Supplementary Table 1. List of genes involved in Parkinson’s disease and other diseases related to parkinsonism included in the four incremented gene panels used in this study**

| **Gene** | **Loci or related diseases** | **NM_transcripts** | **Recessive_only** | **All_V.1** | **All_V.2** | **All_V.3** |
| --- | --- | --- | --- | --- | --- | --- |
| **Recessive PD-associated genes** | |  |  |  |  |  |
| *PRKN* | PARK2 | NM_004562.2 | *PRKN* | *PRKN* | *PRKN* | *PRKN* |
| *PINK1* | PARK6 | NM_032409.2 | *PINK1* | *PINK1* | *PINK1* | *PINK1* |
| *DJ-1* | PARK7 | NM_007262.4 | *PARK7* | *PARK7* | *PARK7* | *PARK7* |
| *ATP13A2* | PARK9/SPG78 | NM_022089.2 | *ATP13A2* | *ATP13A2* | *ATP13A2* | *ATP13A2* |
| *PLA2G6* | PARK14/NBIA2B | NM_003560.2 | *PLA2G6* | *PLA2G6* | *PLA2G6* | *PLA2G6* |
| *FBXO7* | PARK15 | NM_012179.3 | *FBXO7* | *FBXO7* | *FBXO7* | *FBXO7* |
| *DNAJC6* | PARK19 | NM_001256864.1 | *DNAJC6* | *DNAJC6* | *DNAJC6* | *DNAJC6* |
| *SYNJ1* | PARK20 | NM_003895.3 | *SYNJ1* | *SYNJ1* | *SYNJ1* | *SYNJ1* |
| *VPS13C* | PARK23 | NM_020821.2 | *VPS13C* | *VPS13C* | *VPS13C* | *VPS13C* |
| *ADORA1* | PARK | NM_000674.2 |  |  | *ADORA1* | *ADORA1* |
|  |  |  |  |  |  |  |
| **Dominant PD-associated genes** | |  |  |  |  |  |
| *SNCA* | PARK1 | NM_000345.3 |  | *SNCA* | *SNCA* | *SNCA* |
| *LRRK2* | PARK8 | NM_198578.3 |  | *LRRK2* | *LRRK2* | *LRRK2* |
| *VPS35* | PARK17 | NM_018206.5 |  | *VPS35* | *VPS35* | *VPS35* |
| *UCHL1* | PARK5/SPG79 | NM_004181.4 |  | *UCHL1* | *UCHL1* | *UCHL1* |
| *EIF4G1* | PARK18 | NM_182917.4 |  | *EIF4G1* | *EIF4G1* | *EIF4G1* |
| *TMEM230* | PARK21 | NM_001009923.1 |  |  | *TMEM230* | *TMEM230* |
| *DNAJC13* | PARK21 | NM_001329126.1 |  | *DNAJC13* | *DNAJC13* | *DNAJC13* |
| *CHCHD2* | PARK22 | NM_001320327.1 |  |  | *CHCHD2* | *CHCHD2* |
| *TNK2* | PARK | NM_001010938.1 |  |  | *TNK2* | *TNK2* |
| *TNR* | PARK | NM_003285.2 |  |  | *TNR* | *TNR* |
| *RIC3* | PARK | NM_024557.4 |  |  | *RIC3* | *RIC3* |
| *PODXL* | PARK | NM_001018111.2 |  |  | *PODXL* | *PODXL* |
| *LRP10* | PARK | NM_014045.3 |  |  |  | *LRP10* |
|  |  |  |  |  |  |  |
| **Related Parkinsonism-associated genes** | |  |  |  |  |  |
| *SPG11* | PARK/SPG11 | NM_025137.3 |  | *SPG11* | *SPG11* | *SPG11* |
| *GCH1* | PARK_DYT | NM_001024024.1 |  | *GCH1* | *GCH1* | *GCH1* |
| *TH* | PARK_DYT | NM_199292.2 |  | *TH* | *TH* | *TH* |
| *THAP1* | DYT | NM_018105.2 |  |  |  | *THAP1* |
| *POLG* | PARK/Mitochondrial diseases | NM_002693.2 |  | *POLG* | *POLG* | *POLG* |
| *DCTN1* | PARK/Perry syndrome | NM_004082.4 |  | *DCTN1* | *DCTN1* | *DCTN1* |
| *PANK2* | PARK/NBIA1 | NM_153638.2 |  | *PANK2* | *PANK2* | *PANK2* |
| *RAB39B* | PARK/X-linked ID | NM_171998.3 |  |  | *RAB39B* | *RAB39B* |
| *MAPT* | DFT/PSP | NM_001123066.3 |  |  |  | *MAPT* |
| *TARDBP* | DFT | NM_007375.3 |  |  |  | *TARDBP* |
| *VCP* | DFT | NM_007126.3 |  |  |  | *VCP* |
| *GRN* | DFT | NM_002087.2 |  |  |  | *GRN* |
| *PSEN1* | AD/DFT | NM_000021.4 |  |  |  | *PSEN1* |
| *PSEN2* | AD | NM_000447.3 |  |  |  | *PSEN2* |
|  |  |  |  |  |  |  |
| **Risk factor for PD** | |  |  |  |  |  |
| *GBA* | Candidate gene | NM_001005741.2 |  | *GBA* | *GBA* | *GBA* |
| *ACMSD* | GWAS | NM_138326.2 |  |  |  | *ACMSD* |
| *RAB29/NUCKS1* | GWAS | rs823118 |  |  | rs823118 | rs823118 |
| *SIPA1L2* | GWAS | rs10797576 |  |  | rs10797576 | rs10797576 |
| *TMEM163* | GWAS | rs6430538 |  |  | rs6430538 | rs6430538 |
| *STK39* | GWAS | rs1474055 |  |  | rs1474055 | rs1474055 |
| *MCCC1* | GWAS | rs12637471 |  |  | rs12637471 | rs12637471 |
| *TMEM175* | GWAS | rs34311866 |  |  | rs34311866 | rs34311866 |
| *FAM200B* | GWAS | rs11724635 |  |  | rs11724635 | rs11724635 |
| *FAM47E* | GWAS | rs6812193g |  |  | rs6812193 | rs6812193 |
| *SNCA* | GWAS | rs356182 |  |  | rs356182 | rs356182 |
| *HLA-DRB6* | GWAS | rs9275326 |  |  | rs9275326 | rs9275326 |
| *KLHL7* | GWAS | rs199347 |  |  | rs199347 | rs199347 |
| *BAG3* | GWAS | rs117896735 |  |  | rs117896735 | rs117896735 |
| *MIR4697* | GWAS | rs329648 |  |  | rs329648 | rs329648 |
| *LRRK2* | GWAS | rs76904798 |  |  | rs76904798 | rs76904798 |
| *OGFOD2* | GWAS | rs11060180 |  |  | rs11060180 | rs11060180 |
| *GCH1* | GWAS | rs11158026 |  |  | rs11158026 | rs11158026 |
| *VPS13C* | GWAS | rs2414739 |  |  | rs2414739 | rs2414739 |
| *ZNF646* | GWAS | rs14235 |  |  | rs14235 | rs14235 |
| *MAPT* | GWAS | rs17649553 |  |  | rs17649553 | rs17649553 |
| *SYT4* | GWAS | rs12456492 |  |  | rs12456492 | rs12456492 |
| *DDRGK1* | GWAS | rs8118008 |  |  | rs8118008 | rs8118008 |
| *RP1–13N12.1* | GWAS | rs591323 |  |  | rs591323 | rs591323 |
| *SREBF1/RAI1* | GWAS | rs11868035 |  |  | rs11868035 | rs11868035 |
|  |  |  |  |  |  |  |
| **Internal candidate genes for autosomal recessive PD** | | |  |  |  |  |
| *PSMB10* |  | NM_002801.3 |  |  |  | *PSMB10* |
| *SSH2* |  | NM_001282129.1 |  |  |  | *SSH2* |
| *COX5A* |  | NM_004255.3 |  |  |  | *COX5A* |
| *PSME3* |  | NM_176863.2 |  |  |  | *PSME3* |
| *COX19* |  | NM_001031617.2 |  |  |  | *COX19* |
| *NAPRT* |  | NM_145201.5 |  |  |  | *NAPRT* |
| *EPG5* |  | NM_020964.2 |  |  |  | *EPG5* |
| *PSMF1* |  | NM_178578.3 |  |  |  | *PSMF1* |

Nota: All gene sequences were obtained from the latest build of the human reference genome, GRCh38 (for Genome Research Consortium human build 38) or hg38 (for Human genome build 38).
